# Effect of Replacing Cocoa Powder by Carob Powder in the Muffins on Sensory and Physicochemical Properties

**DOI:** 10.1007/s11130-018-0675-0

**Published:** 2018-06-08

**Authors:** Katarzyna Pawłowska, Maciej Kuligowski, Iwona Jasińska-Kuligowska, Marcin Kidoń, Aleksander Siger, Magdalena Rudzińska, Jacek Nowak

**Affiliations:** 1 Faculty of Food Science and Nutrition, Poznań University of Life Sciences, Poznań University of Life Sciences, ul. Wojska Polskiego 31, 60-637 Poznań, Poland; 2 Department of Food Commodity, Faculty of Commodity Sciences, Commodity Science, Poznań University of Economics and Business, al. Niepodległości 10, 61-875 Poznań, Poland

**Keywords:** Carob powder, Cocoa, Phytosterols, Antiradical activity, Isoflavones

## Abstract

The increasing demand for cocoa and search for ingredients rich in bioactive compounds encouraged us to investigate the possibility of replacing it by carob powder in the muffins containing soy beans, sesame oil and flaxseeds. There was 5% addition of carob or cocoa powder to the individual doughs. The muffins with the addition of carob were characterized by improved antiradical activity (by 36% - 2,2′-azino-bis (3-ethylbenzothiazoline-6-sulfonic acid) (ABTS) method, by 83% - 2,2-diphenyl-1-picrylhydrazyl (DPPH) method), higher content of genistein (18%) and total phytosterols (17%) in the dry mass. The color differences in the carob muffins crusts were not perceptible by consumers (ΔE = 0.70 for crust, ΔE = 5.6 for crumb) and their taste was found to be less bitter and sweeter than the taste of cocoa muffins. Moreover, the addition of carob powder as well as cocoa powder resulted in good sensory quality. The high content of phytosterols, genistein and improved antiradical properties proved carob to be a source of bioactive compounds. The results show that carob powder may be used as valuable alternative muffin ingredient to cocoa.

**Electronic supplementary material:**

The online version of this article (10.1007/s11130-018-0675-0) contains supplementary material, which is available to authorized users.

## Introduction

Despite the increasing awareness of the influence of food on health, food-related diseases are still a growing problem in the western world. Cakes and cookies are readily eaten as snacks or desserts. These products generally contain low quality ingredients such as saturated fats and have high energy density, high glycemic response and low satiating effect [1]. Nowadays a lot of time is aimed at investigation of enriching food products in healthy and innovative additives [2].

The growing consumer demand for foods of higher nutritional and sensory quality has encouraged research on new ingredients to be used in bakery products. In this study muffins were enriched with cocoa or carob powder. Apart from these additives several unconventional ingredients of high nutritional value were used to make muffins: soybeans, sesame oil and flaxseeds.

Carob, from the nutritional point of view is a good source of dietary fiber and was successfully used in preparation of gluten-free bread [3]. Carob pods contain small amounts of proteins (3–4%) and lipids (0.4–0.8%) and significant amounts of carbohydrates (76%), mainly soluble ones, such as sucrose [4]. Carob is rich in polyphenols, especially in condensed tannins [5] and gallic acid, which is responsible for gene expression modulating and protecting of colon adenoma cells from the genotoxic impact of H_2_O_2_ [6]_._ In the food industry it is mostly used for gum (carob gum, locust bean gum) production because of galactomannans contained in the endosperm of the seeds. Another product made of seedless carob pods is carob powder. Due to cocoa-like aroma and flavor that carob powder obtains after roasting it shows a great potential to substitute the cocoa [5].

Cocoa powder is one of the most popular additives in bakery industry due to its unique flavor and properties. Cocoa polyphenols have been described as bioactive compounds with antioxidant and anticarcinogenic properties [7, 8]. It was proved that cocoa powder and dark chocolate may positively affect cardiovascular disease (CVD) risk status by modestly reducing LDL-cholesterol (low density lipoprotein) oxidation susceptibility, increasing serum total antioxidant capacity and HDL-cholesterol (high density lipoprotein) concentrations [9]. Soybean was also reported to have positive effect on human health. Soybeans contain bioactive compounds such as isoflavones, which may act similarly to estrogen [10, 11]. Soybean products were proved to reduce the risk of coronary heart disease and its consumption showed protective effects on the risk for endocrine-related gynecological cancers [11].

Because the demand for cocoa is ever growing, it is very likely the production will not be able to keep up. This would lead to the rise of prices of cocoa [12]. Finding a substitute for cocoa is becoming very important. Carob powder produced from seedless pods is suggested as a cocoa alternative [12]. Considering carob as a source of many health beneficial compounds, while cocoa has some limitation in consumption due to the caffeine and theobromine content we decided to investigate how substituting cocoa powder with carob powder affects the sensory perception and color in prepared bakery products. The content of sterols, tocopherols, isoflavones content and antiradical activity were also studied.

## Materials and Methods

### Ingredients for Muffins’ Preparation

All ingredients were purchased in groceries in Poznań, Poland. The following ingredients were used: commercial granulated sugar (Diamant, Środa Wlkp., Poland), soy beans (Bio Planet, China), flax seeds (Bio Planet, India), eggs (Jantex, Środa Wlkp., Poland), wheat flour (Młyn Kostrzyn nad Odrą, Kostrzyn, Poland), sesame oil (Tao Tao, China), carob powder (Bio Planet, Italy), cocoa (Magnetic, Wadowice), baking powder (Gellwe, Zabierzów, Poland), sodium bicarbonate (Dr. Oetker, Gdańsk, Poland).

### Chemicals and Reagents

2,2′-Azino-bis (3-ethylbenzothiazoline-6-sulfonic acid) diammonium salt (ABTS), 6-hydroxy-2,5,7,8-tetramethylchroman-2-carboxylic acid (Trolox), 2,2-diphenyl-1-picrylhydrazyl (DPPH), isoflavone and phytosterol standards were from Sigma (Sigma Chemical Co., St. Louis, MO, USA). Tocopherol homologues were from Calbiochem-Merck Biosciences (Darmstadt, Germany). Methanol and acetonitrile (HPLC purity) were from POCh (Gliwice, Poland). Water was purified with the Milli-Q-system (Millipore, Bedford, USA).

### Muffin Preparation

The composition of muffins had been previously developed in the laboratory and the content of 5% of cocoa or carob powders had been chosen as the most acceptable (among 1, 5, 10 and 15%) in the acceptance test performed by consumers. The ingredient proportions were as follows: soy beans 25.5%, white sugar 19.0%, eggs 14.0%, water 13.0%, sesame oil 9.5%, flax seeds 6.5%, wheat flour 6.0%, cocoa or carob powder 5.0%, baking powder 1 and 0.5% sodium bicarbonate. Initially soy beans were soaked for 12 h and then cooked for 45 min and crushed in a kitchen blender. Flax seeds were ground in a laboratory grinder. Then all the ingredients were mixed in planetary mixer Hendi, model 7 for 6 min at the speed 2 and 9 min at the speed 4 to make a homogeneous mixture. The dough was placed into silicon muffin cups (ø 4.6 cm) and baked for 25 min in the oven preheated to 180 °C. Once baked, the cakes were cooled to room temperature. A fresh product was subjected to sensory and color analysis. The other samples were frozen at −20 °C, lyophilized in a laboratory freeze dryer, crushed and sifted.

### Antiradical Activity

Antiradical activities were measured using the ABTS [13] and DPPH radical scavenging assays [14]. One gram of previously defatted and crushed sample was extracted for 1 h with 70% acetone on a roller shaker. The mixture was centrifuged at 1780 *g* for 5 min. 100 and 30 μL aliquots of each sample extract were added to 3 mL of DPPH and ABTS solution, respectively. The decrease in absorbance was determined spectrophotometrically at 520 nm during 20 min for DPPH and at 735 nm during 6 min for ABTS. Antiradical activities were expressed as Trolox equivalent.

### Isoflavone Extraction and HPLC Analysis

One gram of crushed material was suspended in 80% acetonitrile, stirred intensely for 2 h at room temperature and centrifuged at 1310 *g* for 30 min. The supernatant was collected. The material was reextracted in 10 mL of 80% methanol, mixed for 4 h at 80 °C and centrifuged at 1310 *g* for 15 min. The two supernatants were combined and the solvent was evaporated under vacuum and dried under nitrogen [15]. The residue was diluted to a final volume of 2 mL. The samples were filtered through a 0.45 μm filter.

A Waters 2695 HPLC system equipped with a Waters 2996 photodiode array detector (PDA) (Waters Associated, Milford, MA) was used for analysis of isoflavones. The signal was monitored at 200–600 nm. Separation was conducted on an Alltech Alltima C18 reversed-phase column (4.6 × 250 mm, 5 μm, Alltech Co., Deerfield, IL) with a LiChroCART 4–4 RP-18 (5 μm) guard column (Merck KGaA, Darmstadt, Germany), according to the method of Achouri et al. [16]. The chromatographic analysis was performed with isocratic elution at 1 mL min^−1^ flow rate at a detection wavelength of 254 nm. The column temperature was held constant at 26 °C. Injection volume was 10 μL. Quantitative determination of isoflavones was carried out by comparing the retention times and diode array spectral characteristics with the appropriate standards.

### Lipids Extraction

Lipids were extracted from muffins using the Folch procedure [17].

#### Determination of Tocopherols by Normal Phase Chromatography (NP-HPLC)

Fat extracted from muffins (200 mg) was dissolved in *n*-hexane, and diluted up to 10 mL. Tocopherols were qualitatively and quantitatively identified using a Waters HPLC system consisting of a pump (Waters 600), a fluorimetric detector (Waters 474), an autosampler (Waters 2707), a column oven (Waters Jetstream 2 Plus), and a LiChrosorb Si 60 column (250 × 4.6 mm, 5 μm) by Merck (Darmstadt, Germany). The mobile phase was a mixture of *n*-hexane with *1,4*-dioxane (96:4, *v/v*). The excitation and emission wavelengths were 295 and 330 nm, respectively for the fluorescence detection of tocopherols [18].

#### Sterol Contents

Sterol contents and composition were determined by GC, following the procedure described by the AOCS (American Oil Chemists’ Society) method (Ch6–91:2011) [19]. Briefly, lipids (50 mg) were saponified with 2 mL 1 M methanolic KOH for 18 h at room temperature, then 2 mL of water was added and the unsaponifiables were extracted three times using 3 mL of hexane/methyl *tert*-butyl ether (1:1, *v/v*). The solvent was evaporated under a stream of nitrogen. Dry residues were dissolved in 0.2 mL pyridine and silylated with 0.8 mL of SylonBTZ (Trimethylsilyl N-trimethylsilylacetamidate + N-(Trimethylsilyl)imidazole + Chlorotrimethylsilane). Derivatives of the sterols were separated on a HP 6890 series II Plus (Hewlett Packard, Palo Alto, USA) equipped with a DB-35MS capillary column (25 m × 0.20 mm, 0.33 μm; J&W Scientific, Folsom, CA). Hydrogen was used as the carrier gas at a flow rate of 1.5 mL/min. An internal standard, 5α-cholestane, was used for sterol quantification [18].

#### Sensory Analysis

A 10-member trained panel performed Quantitative Description Analysis (QDA) of muffins in three sessions. During these sessions the panellists developed the sensory descriptive terms. The chosen descriptors for taste are: sweet, bitter, cocoa, beany, unknown; for aroma: cocoa, ginger bread, sesame, nut, pumpernickel; for texture: delicate, spongy, granular, sandy. The ratings were made on a 10-point scale (0 – imperceptible, 10 – very perceptible). The samples were kept in the covered boxes for 1 h before testing and in this form presented to the assessors. Samples were served to the panellists three times during 8 h at room temperature under daylight with a serving of water. Acceptance test of muffins was conducted by 78 volunteers (22 men and 56 women, 20–45 years). Muffins were evaluated day after baking on the basis of overall acceptance according to 9-point hedonic scale ranking from 1 (“dislike extremely”) to 9 (“like extremely”).

#### Color Measurement

The color of the muffin crumb and crust were measured using a Konica Minolta CM-3600d spectrophotometer (Konica Minolta Holdings Inc., Tokyo, Japan). The results were expressed as CIE L*, a*, b* color system coordinates. Measurements were made with a D65 illuminant, 25.4 mm diameter of the measurement hole, 10 standard observers and SCE measurement model. The results represent mean of six measurements at different points of the muffin crumb and crust. The color difference between the samples was calculated according to Eq. (1):

1 $$ \Delta  E=\sqrt{{\left({L}_1-{L}_2\right)}^2+{\left({a}_1-{a}_2\right)}^2+{\left({b}_1-{b}_2\right)}^2} $$

#### Statistical Analysis

All analyses were made in triplicates. Statistical analysis was carried out using Statistica 12 StatSoft software. The Tukey’s multiple means comparison test was used to verify differences between the samples. *P* < 0.05, was set as the criterion of significance.

## Results and Discussion

### Antiradical Activity

The results of antiradical properties are presented in Table 1. Both methods using ABTS and DPPH free radicals showed that the antiradical activity was higher in carob muffins than in cocoa ones (by 36% - the ABTS method, by 83% - the DPPH method). During the cocoa and carob processing, the Maillard reaction contributes to the formation of reducing substances (*e.g.*, melanoidins), which reducing power is responsible for their free-radical scavenging activity increasing the antiradical effect [14]. Results of antiradical activity in method with the ABTS radical were higher than with the DPPH radical, but differences between cocoa and carob muffins were the same – 10 μg of Trolox equivalent. Batista et al. [14] also found that the effect of the reaction with the ABTS radical in chocolate and cocoa beans to be higher than with DPPH radical. Both assays are based on scavenging ability of antioxidants. Products with carob powder addition showed proportional dependencies in counteracting the free radicals measured with ABTS and FRAP (ferric reducing ability of plasma) [20]. Sȩczyk et al. [20] have found siginificantly higher antiradical activity in pasta fortified with carob flour. Therefore carob powder can be considered as a significant source of compounds with the antiradical properties. No information about antiradical properties of carob containing products subjected to baking has been found in the literature. There is one description of short time boiling of pasta enriched with this compound [20]. In our study the reference sample, as well as in the study of Rosa et al. [21] was the product with the addition of cocoa powder. In this study the addition of carob powder increased the antiradical properties of muffins. The total antiradical activity of carob powder increased during roasting [22], while the antiradical properties of roasted cocoa beans varied significantly among the cultivars and geographical regions and were affected by roasting conditions [8]. These factors could influence the antiradical properties of carob and cocoa powders.

**Table 1:** The antiradical activity, tocopherol and isoflavone content in muffins

Component	Cocoa muffin	Carob muffin
Antiradical activity (Trolox μg/100 g DM)		
ABTS	18.43 ± 0.82^b^	28.59 ± 2.24^a^
DPPH	2.11 ± 0.12^b^	12.10 ± 0.13^a^
Tocopherols (mg/100 g DM)		
α-T	1.83 ± 0.06^a^	1.51 ± 0.04^b^
β-T	0.16 ± 0.02^a^	0.17 ± 0.01^a^
γ-T	11.24 ± 0.21^a^	9.23 ± 0.14^b^
δ-T	2.88 ± 0.13^a^	2.47 ± 0.18^b^
Isoflavones (μg/100 g DM)		
Daidzin	1687.4 ± 83.95^a^	1546.5 ± 84.86^a^
Glycitin	0.17 ± 0.02^a^	0.16 ± 0.01^a^
Genistin	3665.2 ± 93.84^a^	3601.7 ± 231.08^a^
Daidzein	534.0 ± 37.72^a^	485.3 ± 17.99^a^
Genistein	396.8 ± 25.96^b^	483.0 ± 13.84^a^

*Values (means ± SDs) bearing different superscripts are statistically significantly different (*P* < 0.05). SDs - standard deviations

### Isoflavone Content

The isoflavone content in the muffins is shown in the Table 1. The carob muffins were characterized by higher genistein content (by 18% in DM). Carob belongs to the *Fabaceae* family [12] but only soybean, among commonly eaten plants, contains significant amounts of isoflavones [10]. The positive influence of isoflavones on health is proved and well documented. Genistein is a promising, potent drug in the prophylaxis and treatment of breast and prostate cancer. It also exhibits health beneficial effects in postmenopausal bone loss and osteoporosis, cardiovascular disorders and menopausal symptoms [23]. Therefore in the study, only isoflavones are investigated despite the presence of other polyphenols in the cocoa and carob. In the scientific literature genistein in carob has been reported only once at the level of 4 μg/g [24]. The ability to identify genistein but not glycoside was likely due thermal processing. Chien et al. [25] have observed the increase of aglycones’ content during heating in 200 °C, especially genistein. Padhi et al. [10] have developed muffins with similar proportions of soy as in this research (24 and 25.4%, respectively), but the content of daidzin in their product was about 10 times lower, whereas the content of genistin was about 16 times lower. The differences may have been caused by various isoflavone amounts in the soybean, which may depend on the variety, region and conditions of growth. Soy isoflavones were found to be bioavailable from those muffins and regular consumption resulted in greater than a threefold increase in plasma isoflavones with a doubling of the dose. Although the main source of isoflavones in the muffins is soybean, carob may significantly influence the genistein content. The combination of flavan-3-ols from cocoa and soy isoflavones have improved biomarkers of CVD risk, highlighting the additional benefit of flavonoids to standard drug therapy in managing CVD risk in postmenopausal type 2 diabetic patients [26]. There are no reports on the positive effect of the combination of carob and soy bioactive compounds on human health.

### Tocopherol Content and Sterol Composition

The concentration of tocopherols in the muffins is shown in Table 1. The statistically different isomers in the muffins were: γ-tocopherol, δ-tocopherol and α-tocopherol (higher by 18, 14, 17% in the cocoa muffins, respectively). It was found that legumes, including soy, are the source of γ-tocopherol. γ-tocopherol exhibits less vitamin E activity than α-tocopherol. However, this isomer has been proven to have other biological activities, such as inhibition of oxidative reactions mediated by NO_2_, potentially offering protection against carcinogenesis [27]. In this study the content of γ- δ- α-tocopherols in the cocoa muffins was significantly higher. The carob was not described as a source of tocopherols [28]. However carob polyphenols have been proved to reduce the loss of tocopherols during processing of sunflower oil and have protected against α-tocopherol oxidation of fish mince, showing a concentration-dependent antioxidant effect [29, 30].

The content of individual sterols in the muffins is shown in Table 2. Results show that predominant component in both types of muffins was cholesterol. The source of it was egg yolk. The muffins also contained phytosterols. Phytosterols bear close structural resemblance to cholesterol, but have different side chain configurations. They have an important biological function as they effectively hamper the absorption of cholesterol [31].

**Table 2:** Sterol content (mg/100 g DM) of cocoa and carob muffins

Sterols	Cocoa muffin	Carob muffin
Cholesterol	98.84 ± 1.48^a^	87.17 ± 0.07^b^
Campesterol	13.83 ± 0.23^b^	15.42 ± 0.24^a^
Campestanol	1.67 ± 0.05^b^	1.91 ± 0.14^a^
D5-Stigmasterol	8.79 ± 0.07^b^	9.30 ± 0.07^a^
β-Sitosterol	36.21 ± 0.56^b^	40.85 ± 0.04^a^
Sitostanol	1.51 ± 0.07^a^	1.53 ± 0.12^a^
D5-Avenasterol	4.56 ± 0.10^b^	5.42 ± 0.11^a^
Gramisterol	1.29 ± 0.07^b^	1.49 ± 0.08^a^
D7-Stigmasterol	5.27 ± 0.01^b^	5.92 ± 0.10^a^
Cycloartenol	0.68 ± 0.02^a^	0.71 ± 0.02^a^
D7-Avenasterol	0.58 ± 0.08^b^	0.76 ± 0.02^a^
24-Methylenecycloartanol	1.30 ± 0.03^b^	1.61 ± 0.00^a^
Citrostadienol	n.d.	6.01 ± 0.23^a^
Total phytosterols	75.69 ± 10.27^b^	90.94 ± 11.35^a^
Total sterol	174.53 ± 27.48^b^	178.11 ± 24.60^a^

n.d.– not detected

*Values (means ± SDs) bearing different superscripts are statistically significantly different (P < 0.05). SDs - standard deviations

The content of phytosterols in the carob muffins was significantly higher than in the cocoa muffins (by 17%) (Fig. S1). There are no reports about phytosterol content in the carob. The most abundant phytosterol were β-sitosterol and campesterol.

### Color Parameters

The color parameters are shown in the Table 3. The lightness of carob muffin crust was similar to cocoa muffin. The carob muffin crumb was lighter compared to cocoa muffins’ crumb by 5.39 (L*). The value of a* parameters was similar in both muffins. The same situation was observed in case of b* parameters. The value of a* parameters indicates redness when positive or greenness when negative, while b* indicates yellowness when positive or blueness when negative. All values were close to 0. This means that the color of the muffins did not have an apparent hue, referring to achromatic grey shadow. Also, color differences (ΔE) between the muffins with carob and cocoa were calculated. As far as the external layer (crust) of the muffins is concerned, the difference was not greater than 1, so it was not noticeable for an average observer. However, the color difference between the crumbs was more than 5 - the difference the human eyes can detect and identify. Maillard reaction products were the most responsible for the color formation of the muffins. To conclude, cocoa powder can be replaced by carob powder, causing only minor visible changes to the crumb. Apart from that, the color difference in the crust is not noticeable for regular consumers. The final color of the bakery products containing carob powder can be adjusted by choosing appropriate level of roasting [22]. Rosa et al. [21] have obtained results indicating significant changes in colors in the cakes with banana and soy flours, in which cocoa powder was replaced by carob powder. However, analysis of cakes with carob powder made by Rosa et al. [21] has shown that they came out with a darker color, which was opposite to our observations.

**Table 3:** The color parameters CIE L*a*b* of muffins

Parameters	CCM crust	CRM crust	CCM crumb	CRM crumb
L*(D65)	23.25 ± 0.24^a^	23.89 ± 0.18^a^	19.38 ± 1.95^b^	24.77 ± 0.63^a^
a*(D65)	1.97 ± 0.20^b^	2.12 ± 0.09^a^	2.69 ± 0.45^a^	3.00 ± 0.21^a^
b*(D65)	0.82 ± 0.10^b^	1.03 ± 0.13^a^	1.20 ± 0.37^b^	2.76 ± 0.27^a^
ΔE	0.7		5.6	

L* - lightness of the sample (0 = black, 100 = white); a* - redness when positive or greenness when negative; b* - yellowness when positive or blueness when negative; ΔE- the difference between colors

CCM – cocoa muffin, CRM – carob muffin

*Values (means ± SDs) bearing different superscripts are statistically significantly different (*P* < 0.05). SDs - standard deviations

### Sensory Analysis

**Fig. 1:**
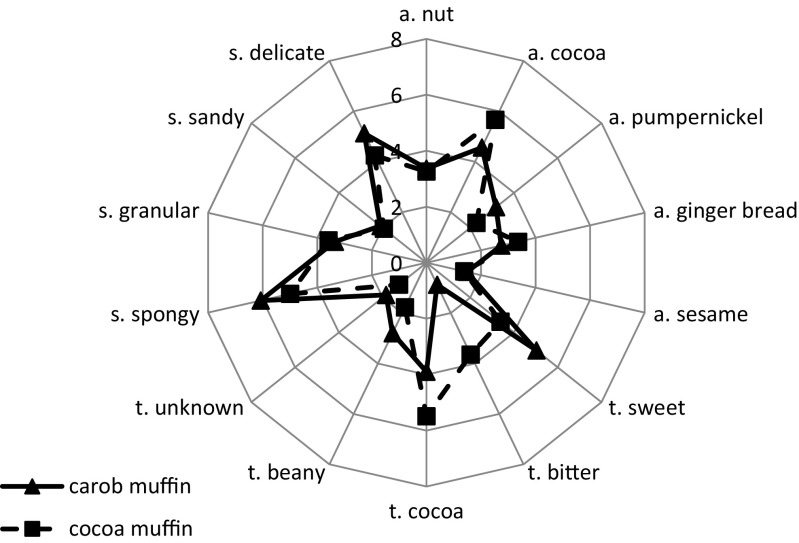
Radar plots of QDA results of cocoa and carob muffins (a-aroma, t-taste, s-structure)

Descriptive sensory analysis (QDA) results of the muffin samples in the radar plot form are shown on Fig. 1. The panel described the samples with 14 sensory terms and statistically significant differences were found. The cocoa and gingerbread aromas were found to be stronger in the cocoa muffins while aroma of pumpernickel was stronger in the carob muffins. In both types of muffins the nut and sesame aromas were characterized by low perceptibility.

The taste of the carob muffin was found to be sweeter, more beany or unknown, and less bitter than the taste of the cocoa muffins. However, the taste of cocoa was stronger in the cocoa muffins. It is also claimed that it may be substituted for up to 30% or even 75% of cocoa in certain products without a perceivable change in flavor [21, 32].

The structure of the carob muffins was characterized as more spongy and delicate than the texture of the cocoa muffins. This may have been caused by the fact that carob powder contains caroubin – a protein with similar properties to gluten, which may facilitate the creation of dough in gluten-free breads [3, 33]. In both types of muffins the texture indicators: sandy and granular were found to be low. However Sęczyk et al. [20] have observed the deterioration of texture in the pasta fortified with carob powder.

**Fig. 2:**
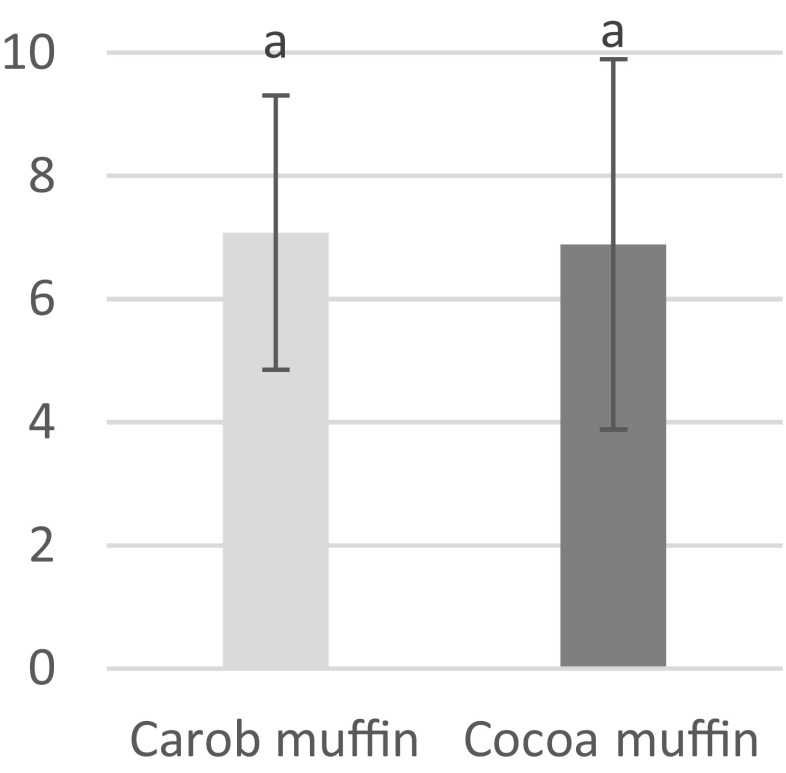
Acceptance test for the carob and cocoa muffins in a 9-point hedonic scale ranking from 1 (“dislike extremely”) to 9 (“like extremely”). Values bearing different superscripts are statistically significantly different (*P* < 0.05)

For general acceptance, carob muffins scored values 7.1 and cocoa muffins 6.9 and no statistically significant differences were found among them (Fig. 2). Although panelists found differences in the perceptibility of aroma, taste and texture descriptors, it did not have any influence on overall acceptance of consumers. In the available literature there is no information about muffins enriched in carob powder which were evaluated by the European’s communities. However there are studies conducted in Canada [34] and Brazil [21] where the products enriched in carob powder obtained lower consumers acceptance. Sensory attributes of carob fortified pasta evaluated by polish consumers were comparable with the reference pasta [20].

## Conclusions

Bakery products are one of the most important dietary components for people all over the world. Therefore, muffins with increased nutritional value were developed. The antiradical activity, isoflavone, tocopherol and sterol content, color and sensory analysis were performed to investigate differences in muffins with the addition of cocoa or carob powder. Both types of muffins showed dominant of cocoa aroma and spongy structure. However, the carob muffins were found to be sweeter. The antiradical activity, the content of genistein and phytosterols in the carob muffins were significantly higher than in cocoa muffins. Furthermore, the addition of carob to muffins resulted in good sensory quality. It shows that carob may be successfully used in bakery products as cheaper cocoa substitute. The replacement of cocoa powder by carob powder in soy muffins represents a technological solution which could lead to reduction of white sugar in bakery products and the production of innovative functional products. Carob can be considered as a valuable additive containing bioactive compounds to produce healthier sweets.

## Electronic supplementary material

Fig. S1 (DOC 62 kb)
