# Supplementary material for: Effect of Replacing Cocoa Powder by Carob Powder in the Muffins on Sensory and Physicochemical Properties
Source: Plant Foods Hum Nutr. 2018 Jun 8;73(3):196–202. doi: 10.1007/s11130-018-0675-0 (PMC6096888; doi:10.1007/s11130-018-0675-0)

**Article Title:** Effect of replacing cocoa powder by carob powder in the muffins on sensory and physicochemical properties

**Journal:** Plant Foods for Human Nutrition.

**Authors:** Katarzyna Pawłowska, Maciej Kuligowski, Iwona Jasińska-Kuligowska, Marcin Kidoń, Aleksander Siger, Magdalena Rudzińska and Jacek Nowak

**Corresponding author:** Katarzyna Pawłowska, katpaw@up.poznan.pl, tel. +48 61 848 7294, fax +48 61 848 73 14, Faculty of Food Science and Nutrition, Poznań University of Life Sciences, Poznań University of Life Sciences, ul. Wojska Polskiego 31, 60-637 Poznań, Poland

**Fig. S1** GC-FID chromatogram of sterols for the carob muffin.


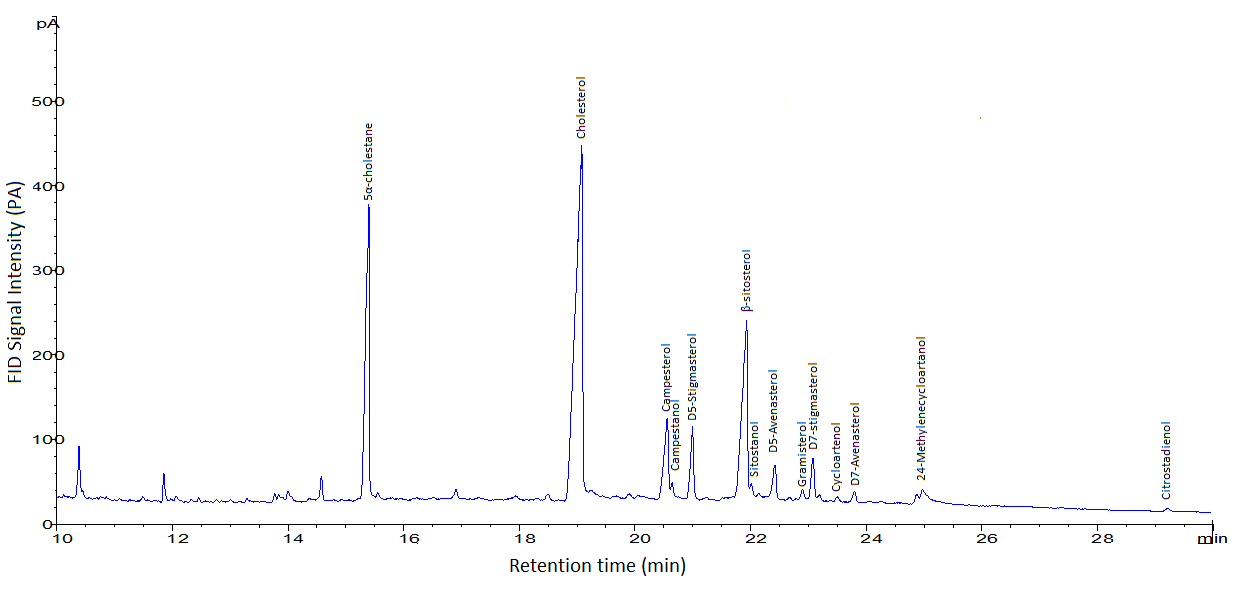

Supplement: Supplementary file 1 — (DOC 62 kb) [file 11130_2018_675_MOESM1_ESM.doc]
